# Mantle cell lymphoma first presenting as immune complex-mediated glomerulonephritis: a case report

**DOI:** 10.1186/s13256-015-0583-y

**Published:** 2015-05-19

**Authors:** Rajitha Asanga Abeysekera, Abdul Wahid Mohomad Wazil, Nishantha Nanayakkara, Neelakanthi Ratnatunga, Kaushal Maithree Fernando, Jalitha Thinnarachchi

**Affiliations:** 1 Nephrology and Transplantation Unit, Teaching Hospital, Kandy, Sri Lanka; 2 Faculty of Medicine, University of Peradeniya, Peradeniya, Sri Lanka

**Keywords:** Immune complex glomerulonephritis, Mantle cell lymphoma, Non-Hodgkin lymphoma

## Abstract

**Introduction:**

Kidney involvement in non-Hodgkin lymphoma is recognized but mostly diagnosed following a diagnosis of lymphoma. We describe a rare case of mantle cell lymphoma, a B-cell-type non-Hodgkin lymphoma, first presenting with immune complex glomerulonephritis.

**Case presentation:**

We report the case of a 58-year-old Sri Lankan man who presented with gross hematuria. Further investigation revealed bicytopenia with splenomegaly and elevated serum creatinine. He had a renal biopsy, which revealed acute immune complex glomerulonephritis with interstitial inflammation. Results from an initial bone marrow biopsy and blood imaging were inconclusive. Three months later his renal function had deteriorated and a lymph node biopsy revealed mantle cell lymphoma. Within three months of initiating chemotherapy, his renal function returned to normal levels and remained normal at one year of follow-up.

**Conclusions:**

It is important to have a high degree of suspicion when patients present with acute immune complex glomerulonephritis with no other identifiable cause, because it could be the first presentation of a non-Hodgkin lymphoma such as mantle cell lymphoma.

## Introduction

The kidney is a fascinating organ that, with its complex architecture and physiology, can be the first site of clinical disease manifestation in many systemic disorders. Systemic disorders such as vasculitic disorders, connective tissue disorders, hematological malignancies, and many solid organ malignancies, as well as systemic infections, are known to have renal manifestations. Similarly, non-Hodgkin lymphoma (NHL) can also have renal involvement. Over the last few decades the incidence and survival of patients with lymphomas, especially NHL, have increased, making its different pathological manifestations more important [1]. Kidney involvement can occur in many ways in NHL. Symptoms can be related to obstruction, drug-related nephrotoxicity, direct infiltration of NHL, or development of primary renal lymphomas; kidney involvement can also manifest as a paraneoplastic syndrome [1].

We describe a rare case of B-cell NHL mantle cell lymphoma (MCL) that first presented with acute immune complex-mediated glomerulonephritis.

## Case presentation

A 58-year-old previously well Sri Lankan man was admitted to our general medical unit with two episodes of gross hematuria with no frothy urine, dysuria, colicky abdominal pain or reduction in urine output. He had no preceding history of illness, fever, any other bleeding diathesis, or any history suggestive of a connective tissue disease. Our patient was mildly pale with mild splenomegaly and an elevated blood pressure of 150/90mmHg, but rest of the physical examination was normal. Initial laboratory investigations revealed an elevated serum creatinine of 2.9mg/dL (normal range: 0.7 to 1.3mg/dL). A urinary microscopic examination was significant with 100 to 120 red cells per high power field: 30% of cells were dysmorphic and his protein level was 600mg/dL. His blood leucocyte count was 5800/μL (normal range: 4000 to 11,000/μL), hemoglobin was 8.6g/dL (normal range: 11 to 16g/dL), and platelet count was 124,000/μL (normal range: 150,000 to 450,000/μL). His erythrocyte sedimentation rate was 20mm/h, his level of C-reactive protein was 1.1mg/L (normal range: 0 to 5mg/L), and serum albumin was 3.3g/dL (normal range: 3.6 to 5.5g/dL).

**Figure 1:**
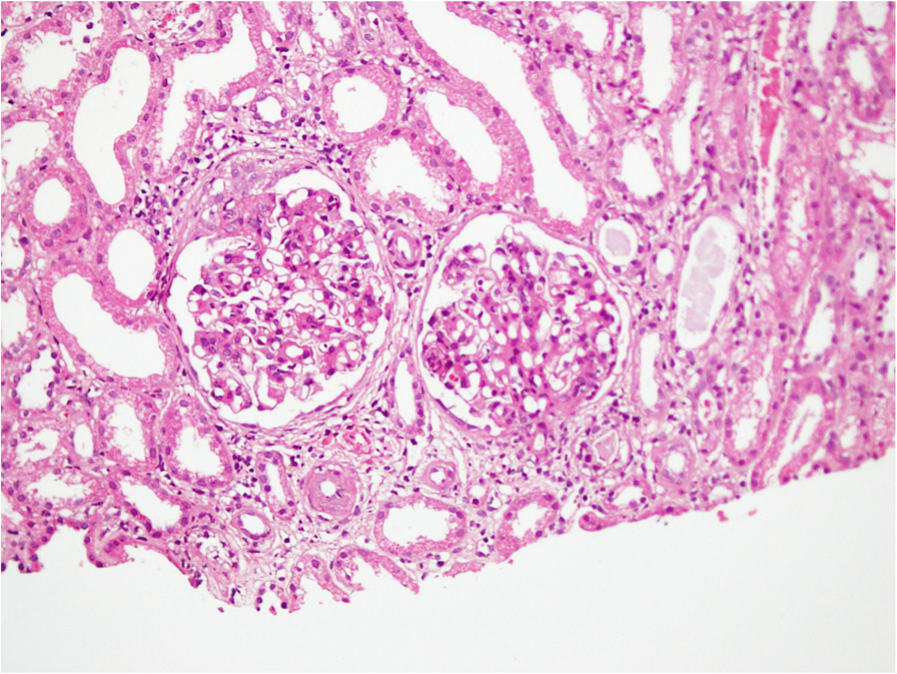
Renal biopsy (×200 magnification). Diffuse increase in mesangial cells and matrix. Occasional neutrophils seen. Occasional foci of endocapillary proliferation seen. Focal parietal epithelial cell hyperplasia with occasional tuft adhesions. Capillaries are thickened but no double contouring or spikes were seen.

**Figure 2:**
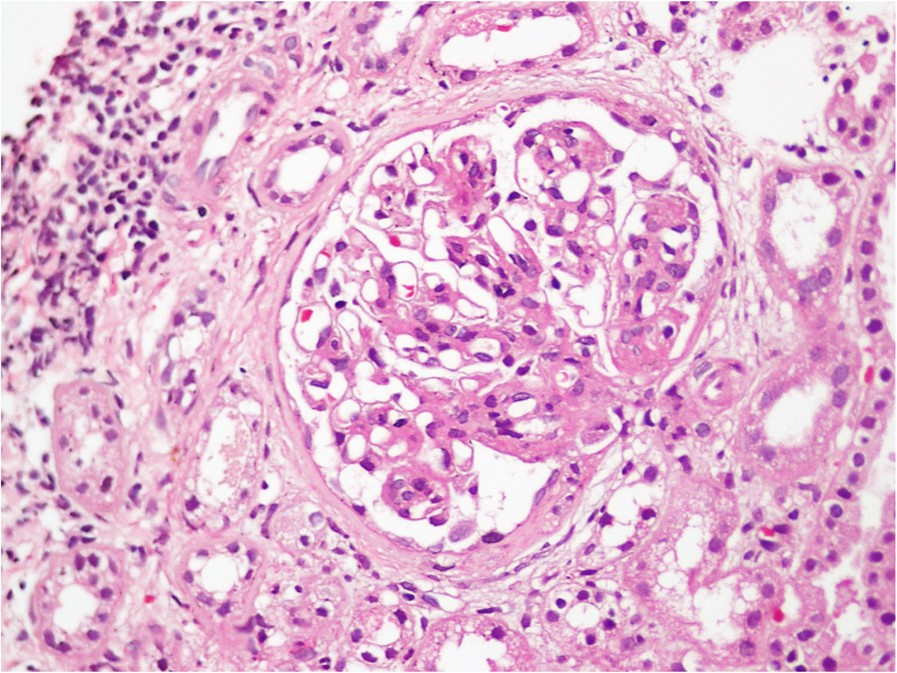
Renal biopsy (×400 magnification). Focal interstitial lymphocytic infiltrate present. Focal tubular atrophy and interstitial fibrosis with periglomerular fibrosis in these foci are seen. Red cells, protein, and granular casts are seen.

An ultrasound examination of our patient’s abdomen revealed normal-sized kidneys with increased echogenicity with mild splenomegaly.Blood film was reported as suggestive of anaemia of chronic disorder. In view of the bicytopenia and mild splenomegaly with significant hematuria, we performed a renal biopsy and a bone marrow biopsy. Results from the bone marrow biopsy showed no evidence of marrow infiltration by leukemia, lymphoma, myeloma or secondary deposits. The specimen from the renal biopsy (Figures 1 and 2) had 14 glomeruli, seen on the formalin-fixed paraffin sections. The glomeruli showed a mild diffuse increase in mesangial cells and matrix, and occasional tuft adhesions. Occasional foci of endocapillary proliferation were seen. The capillary basement membranes were normal. There were no crescents. We found focal infiltrates of lymphocytes in the interstitium. There were red cell and granular casts. We also noted occasional foci with tubular atrophy, interstitial fibrosis, and periglomerular sclerosis.

Eight glomeruli were seen on frozen sections for immunofluorescence studies. Direct immunofluorescence staining showed fine granular deposits of immunoglobulin (Ig) G (3+) and complement 3 (4+) in capillaries in all glomeruli, and IgM (2+) in the capillaries and mesangium in two glomeruli segmentally. There was no positive staining for IgA . We did not perform electron microscopy because it was not available at our institution. Overall, this renal histology was consistent with immune complex-mediated acute glomerular nephritis.

Following this initial presentation our patient defaulted on follow-up. Three months later, he presented with bilateral ankle edema with periorbital swelling. He also complained of loss of appetite but had no other constitutional symptoms. An examination revealed mild pallor, a left-side discrete axillary lymph node, and moderate splenomegaly.

Investigations demonstrated worsening of his renal function, with serum creatinine of 3.18mg/dL and a urine protein to creatinine ratio of 1432mg/g. A urinary microscopic examination revealed 180 to 200 red blood cells per high power field, of which 40% were dysmorphic, along with red cell casts and coarse granular casts. An ultrasound examination of his kidneys revealed normal-sized kidneys (left 13.1cm; right 11.1cm) with increased echogenicity. His blood leucocyte count was 2900/μL, hemoglobin was 8.8g/dL, and his platelet count was 104,000/μL. Serologic test results for hepatitis B and C, and human immunodeficiency virus were negative, as was a Venereal Disease Research Laboratory test. Tests for antinuclear antibody and antineutrophil cytoplasmic antibody were negative. His complement levels for both C3 and C4 were normal, with a C3 level of 115.2mg/dL (normal range: 90 to 180mg/dL) and a C4 level of 27mg/dL (normal range: 10 to 40mg/dL). Serum cryoglobulins were not detected. Serum protein electrophoresis did not show evidence of a monoclonal gammopathy and myeloma screening was negative. His lactate dehydrogenase level was 404.9U/L.

**Figure 3:**
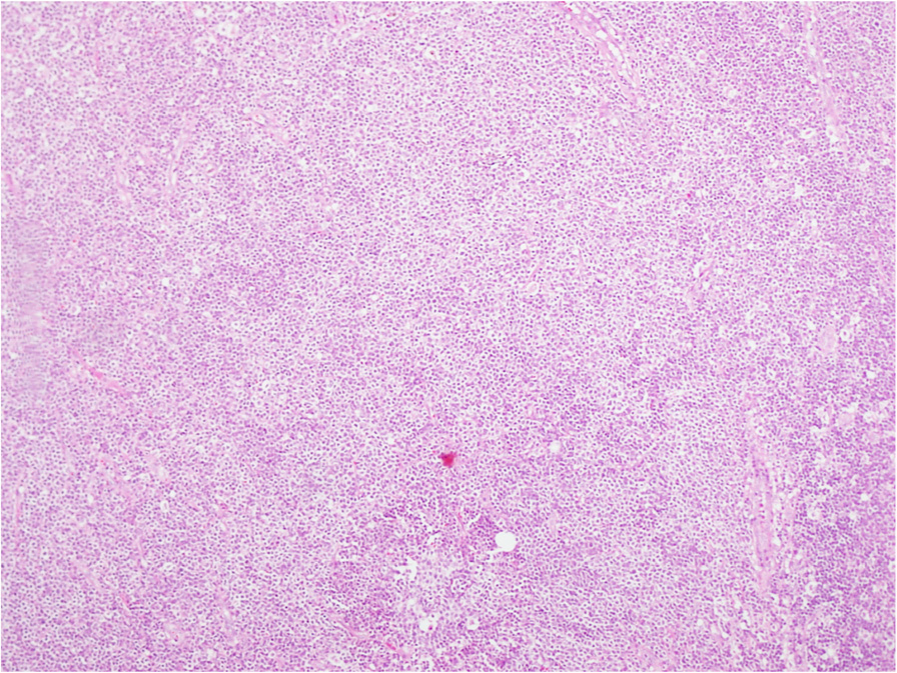
Lymph node biopsy (×100). Sections of the lymph node showed an effaced architecture, and were diffusely infiltrated by a monotonous population of small lymphoid cells.

**Figure 4:**
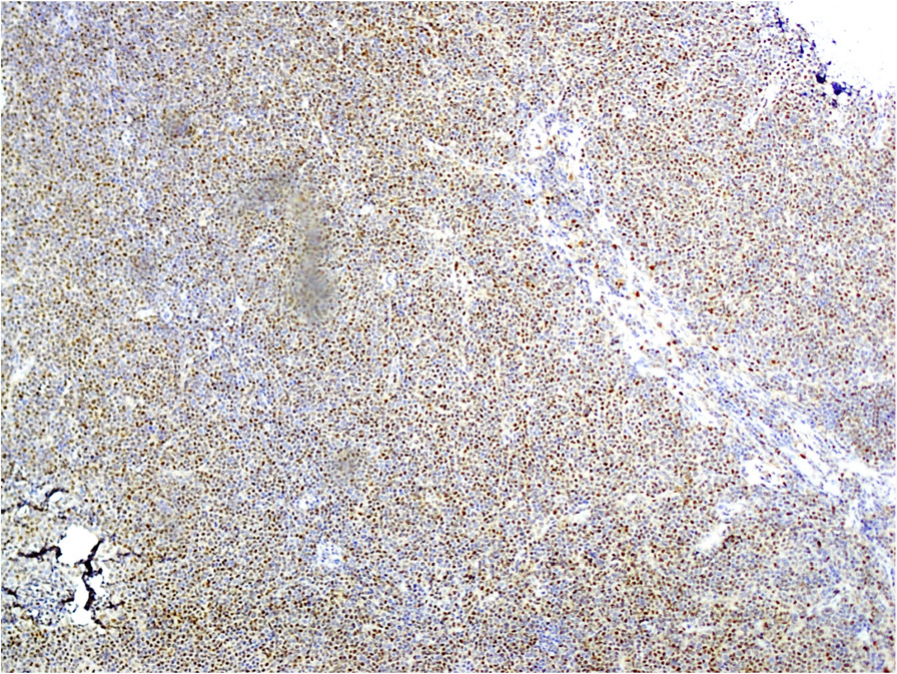
Lymph node biopsy (×100). Cyclin D1 was strongly expressed in >90% of tumor cells.

**Figure 5:**
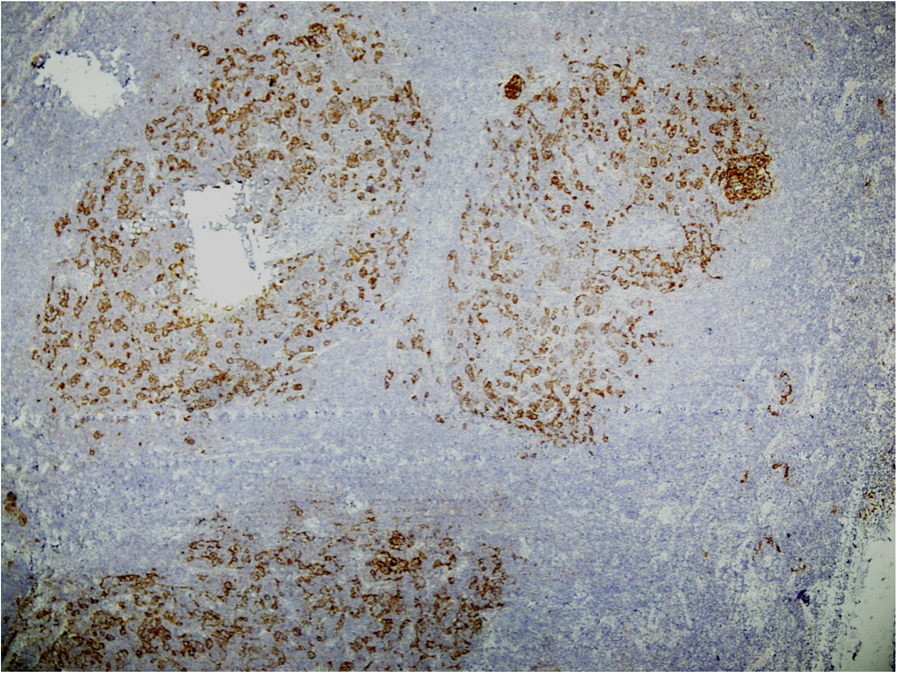
Lymph node biopsy (×140). CD23 stain highlighted nodular aggregates of follicular dendritic cells. The tumor cells were negative for CD23.

We performed an axillary lymph node biopsy, which showed sections of his lymph node with an effaced architecture, diffusely infiltrated by a monotonous population of small lymphoid cells (Figure 3). These cells showed strong membrane staining with CD20. Cyclin D1 was strongly expressed in >90% of the tumor cells (Figure 4). His Ki67 index was 10%. A CD23 stain highlighted nodular aggregates of follicular dendritic cells (Figure 5). The tumor cells were negative for CD23. These findings led to a diagnosis of B-cell NHL MCL.

Therefore a final diagnosis of NHL with immune complex-mediated glomerulonephritis was made and our patient referred for further oncological management.

His oncological management involved a regimen of rituximab, cyclophosphamide, doxorubicin, vincristine, and prednisolone (R-CHOP). Follow-up at three months showed complete normalization of his renal function to a serum creatinine of 1.2mg/dL (normal range: 0.7 to 1.3mg/dL) and a normal urine full report (protein - trace; red cells - nil). One year after chemotherapy, his renal function remained normal (serum creatinine 1.0mg/dL) with a normal full blood count (white cell count 6100/μL, hemoglobin 13.2g/dL, platelet count 234,000/μL) and a serum albumin level of 3.9g/dL.

## Discussion

Lymphomas are a type of tumor involving the immune system, of which NHL constitutes around 90% [2]. The other 10% consists of Hodgkin lymphoma. Lymphoma is the fifth most frequently diagnosed cancer in the UK [2]. According to the most recent World Health Organization classification of tumors of hemopoietic and lymphoid tissues published in 2008, NHLs are broadly divided into B-cell, T-cell and natural killer-cell lymphoma [2]. MCL is a distinct subtype of B-cell NHL. It is recognized as a highly aggressive lymphoma subtype that can present very late in its disease course [3].

Glomerulonephritis (GN) in NHL is a rare phenomenon and the type of disease can vary widely [4]. Minimal change disease, focal segmental glomerulosclerosis, membranous GN, membranoproliferative GN, mesangioproliferative GN, IgA nephropathy, crescentic GN and fibrillary GN are some of the histopathological types that have been reported in the world literature [5,6]. Considering this heterogeneous morphology, GN poses a great diagnostic challenge for the clinician. Clinical presentation can vary markedly, to include proteinuria, microscopic hematuria, impaired renal functions, acute kidney injury, or rapidly progressive GN [6]. Even though it is common for renal disease to manifest after a diagnosis of NHL, some studies have shown renal involvement to be the first manifestation identified [6].

In our report, we describe a rare case of B-cell NHL MCL where the first presentation for medical attention was because of renal involvement with hematuria. When our patient first presented, he had no significant lymphadenopathy and a bone marrow biopsy did not reveal any evidence of NHL, which may have been owing to the early stage of the disease or patchy involvement of the bone marrow. The clinical picture with lymphadenopathy only manifested three to four months after the renal manifestation. In the absence of other supporting evidence at initial presentation, results from a renal biopsy are very difficult to interpret and mimic resolving acute GN, which is in keeping with the clinical picture. Furthermore, the findings from our patient’s renal biopsy with its immunofluorescence pattern did not conform to any classic glomerular pathological type but showed definitive evidence of proliferative lesions with evidence of immune-mediated pathology. The axillary lymph node biopsy was the definitive diagnostic investigation to confirm a diagnosis of MCL with associated immune complex GN.

The rapidity with which our patient’s renal function and urine sediment normalized within three months of starting chemotherapy suggests a paraneoplastic renal manifestation of MCL. One could hypothesize that the resolution of the immunological process with chemotherapy also resulted in the resolution of the immunological process within the kidney.

There are only a handful of reported cases of MCL with GN; our case is unusual because the first clinical presentation was due to renal involvement. MCL is a rare, aggressive NHL; the few reported cases with GN have described proliferative GN, focal segmental glomerulosclerosis, MCL infiltration with AKI, and AKI due to tubulointerstitial infiltration of MCL [7]. The genetic hallmark of MCL is the chromosomal translocation t(11;14), resulting in aberrant expression of cyclin D1 [8]. The underlying pathogenesis of NHL-associated GN is poorly understood. The focus is currently on immune complexes containing tumor antigens, which are deposited in the glomeruli [9]. Some literature even questions whether this glomerular involvement is paraneoplastic in origin or results from a concurrent primary glomerular disease [10].

Studies regarding the treatment of NHL-associated GN are limited. Current therapeutic management is therapeutic ablation of the NHL, which will lead to spontaneous resolution of the underlying GN [11]. In the management of MCL, multiple chemotherapeutic regimens have been used, including R-CHOP; R-bendamustine; and hyper-fractionated cyclophosphamide, vincristine, doxorubicin, and dexamethasone (Hyper-CVAD) [3].

## Conclusions

It is important to have a high degree of suspicion when patients present with acute immune complex GN with no other identifiable cause because it could be the first presentation of a NHL such as MCL. Although rare, screening for these malignancies is essential in the management of these patients.

## Consent

Written informed consent was obtained from the patient for publication of this case report and accompanying images. A copy of the written consent is available for review by the Editor-in-Chief of this journal.

## Abbreviations

GN: glomerulonephritis
Ig: immunoglobulin
MCL: mantle cell lymphoma
NHL: non-Hodgkin lymphoma
R-CHOP: rituximab, cyclophosphamide, doxorubicin, vincristine, prednisolone
